# Dying within dyads: Stress, sense of security and support during palliative home care

**DOI:** 10.1371/journal.pone.0257274

**Published:** 2021-09-14

**Authors:** Maria Liljeroos, Per Milberg, Barbro Krevers, Anna Milberg

**Affiliations:** 1 Department of Health, Medicine and Caring Sciences, Linköping University, Linköping, Sweden; 2 Centre for Clinical Research Sörmland, Uppsala University, Eskilstuna, Sweden; 3 IFM Biology, Linköping University, Linköping, Sweden; 4 Department of Advanced Home Care and Department of Medical and Health Sciences, Linköping University, Norrköping, Sweden; University of Bologna, ITALY

## Abstract

**Objectives:**

To examine similarities and dissimilarities in patient and family caregiver dyads in their experience of stress, support, and sense of security.

**Methods:**

144 patients and their family caregivers participated. Patients were admitted to six Swedish specialist palliative home care units and diagnosed with a non-curable disease with an expected short survival. We analysed similarity patterns of answers within dyads (correlations) as well as dissimilarities, expressed as the difference between within-dyad responses. The latter were subjected to a model-building procedure using GLM, with 13 sociodemographic and clinical characteristics as independent variables.

**Results:**

Within dyads, patients and family caregivers scored similar in their perception of support and sense of security with care. There was also dissimilarity within dyad responses in their perception of stress and support that could be attributed to sociodemographic or clinical characteristics. When patients scored higher levels of stress than family caregivers, the family caregiver was more likely to be male. Also family caregiver attachment style (attachment anxiety), patient age and the relationship of the family caregiver to the patient explained dissimilarities within the dyads.

**Conclusions:**

Patients and family caregivers within the dyads often, but not always, had similar scores. We suggest that it is important that the healthcare staff identify situations in which perceptions within the dyads regarding stress and perception of support differ, such that they can recognise patients’ and family caregivers’ unique needs in different situations, to be able to provide adequate support and facilitate dyadic coping.

## Introduction

Living with cancer and other serious illnesses is a challenge for the patient and, not least, for the family caregiver. Such a situation also affects the patient and caregiver as a dyad. The concept ‘dyad’ can be defined as a situation in which two individuals maintain a sociologically significant relationship [1,2]. The adjective ‘dyadic’ describes the interaction between the individuals. The strength of a dyadic relationship is built on the basis of the time that the individuals spend together, and on the emotional intensity of their relationship [3]. A caring and warm relationship provides calm and steadiness and can positively influence health outcomes, while a distressed relationship can have a negative impact on health outcomes [4].

Previous research in the context of cancer and other serious illnesses has shown that such dyadic interactions affect both patients’ and family caregivers’ experiences of stress, support and sense of security [5–9]. Thus, good interaction between patients and care staff, including a high-quality care process, ought to improve patients’ sense of security in care.

Today, when hospital stays tend to be shorter, patients increasingly cope with long-term illness in home-based settings, home-based palliative care offers many benefits. It can provide a sense of normalcy and be more comfortable. Further, being able to continue previous activities may facilitate the dyad’s coping to a certain extent [10,11]. Despite being a favourable model of care for dyads who prefer to receive care at home, it may be difficult to provide optimal care in such a model. For instance, palliative home care often relies on a contribution from family caregivers to make it possible [11]. This increases the responsibility placed on the caregiver and may increase the burden on him or her [12,13]. Caregiver burden refers to physical, emotional, social, and financial burden perceived by a caregiver as a result of caring for a sick family member. A high caregiver burden may impair caregivers’ physical and psychological health [14,15] and decrease quality of life [16]. A systematic review including 1233 family caregivers to older cancer patients found that that younger caregivers, solid tumours, and assistance with patient’s activities of daily living and time spent caring for a sick person were significantly associated with high caregiver burden [17]. Also being a spousal caregiver and with lower social support, fewer psychological resources, or less confidence in caregiving have been found to increase the risk of experiencing caregiver burden [18].

A recent review concluded that patients and their family caregivers have unmet needs in the psychosocial domain during home-based palliative care, while physical needs are met [8]. Communication with health professionals was the most frequently reported unmet need for both patients and carers, which contributed to stress and a lack of security [8]. Ellington et al studied communication patterns across the cancer home hospice trajectory and found that as patients decline, caregivers are establishing their individual relationship with nurses and begin to take on the patient’s care management, partnering with the nurse until the end of life [19].

Dyadic interactions in the context of cancer and other serious illnesses are important in relation to the experiences of stress, support and sense of security of patients and caregivers. However, few quantitative studies of dyadic interactions have been carried out for palliative home care. Some studies have been conducted in other contexts, for example in hospital-based palliative care [20–23], and some have used a qualitative approach [24,25].

The aim of the present study was to examine the patterns of perception in patient and family caregiver dyads in their experience of stress, support and sense of security in specialist palliative home care. More specifically, we considered correlations between the responses of patients and family caregivers. Furthermore, we used the difference in responses within dyads to highlight dissimilarities and related these to sociodemographic data and such describing social situation and health.

Based on previous research on dyads within the context of cancer and other serious illnesses, we expected that there would be within-dyad correlations in responses that reflect (i) stress [5,26], (ii) support [25,27], and (iii) sense of security [9], while we are unaware of any previous, relevant studies regarding within-dyad dissimilarities.

## Methods

### Participants and setting

**Fig 1:**
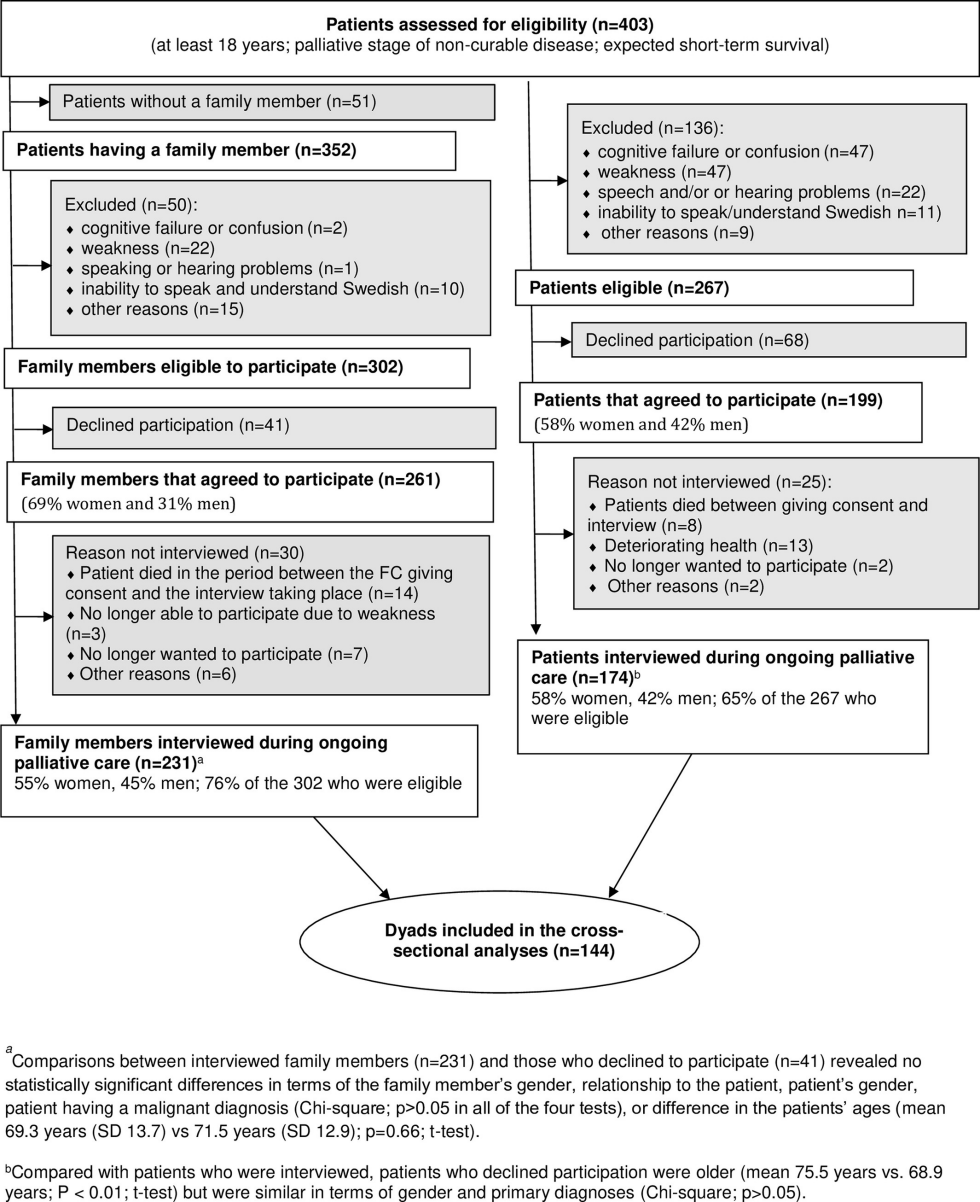
Flow diagram.

In this prospective study, patients suffering from a non-curable disease with an expected short survival (median time for survival 4 months) and their family caregivers were recruited from six specialised palliative home care units in two south-eastern counties of Sweden. Three of the units employed advanced multi-professional palliative home care teams including a physician, specialist nurses, physiotherapist, 24-hour services, and access to a backup ward. The other three units were primary care services with a palliative care consultant and a specialist nurse available during the day. The family caregiver was defined as the person whom the patient had designated for the staff as being his/her family caregiver. This person had been noted in the patient’s medical records (Fig 1).

Fig 1 presents the inclusion and exclusion criteria. Further details of the data collection have already been published [27–29]. The regional board of ethics (”Regionala etikprövningsnämnden i Linköping, Avdelningen för prövning av övrig forskning”) approved the study (Dnr:144–06 (2007-02-09), Dnr:144:06 T100 (2007-12-11); Dnr 2010/278-31 (2010-12-14). Written informed consent was obtained from all individuals included in the study.

### Procedure

During the data collection, 403 patients were admitted to the participating palliative care units, 352 of them with a family caregiver. In total, 174 patients (65% of the 267 who were eligible) and 231 family caregivers (76% of the 302 who were eligible) were interviewed during palliative home care. In these interviews, there were 144 dyads (i.e. a pair that included the patient and his/her family caregiver) who were included in the current analysis. Most of the participants preferred to be interviewed over telephone. During the interviews, dyads answered questions regarding demographics, perceived stress, attachment security, support and sense of security. The questions originated from established instruments (PSS, GSE, COPE, SEC-P, SEC-R, see Table 1 for more detail). With our aims, and the fragile study group involved, it was not possible to use several complete instruments. Instead, we selected parts of instruments deemed most relevant to our aims. Sample size was defined by the number of patients at the six unit, and the length of the sampling period. The data were collected between September 2009 and October 2010. Due to limited research time (teaching and clinical work), this manuscript turned out slow in the making.

**Table 1:** Overview of measured variables regarding patients’ and family caregivers’ experiences of stress, support and sense of security during palliative care asked during the interview.

Main Variables	Measures	Reference no
Sense of security with care^1^	Assessed with two (*Care interaction*; *Mastery*) of three validated subscales from The sense of security in care-Patients’ Evaluation instrument (SEC-P; 15 items) and The sense of security in care-Relatives’ Evaluation instrument (SEC-R; 17 items). Care interaction (SEC-P 3 items, SEC-R 4 items) and Mastery (8 items both from SEC-P and SEC-R); both 6-point scale: 1 (never) - 6 (always); (higher scores representing higher sense of security), mean values of subscales	[28,29]
Perceived stress^1^	*Perceived stress*: Two (of ten) items from the Perceived Stress Scale (PSS) (felt nervous and stressed; difficulties were piling up so high that you could not overcome them); 5-point scale: 0 (never)– 4 (very often); (higher scores representing worse perceived stress), mean value	[30]
	*Self-efficacy*: One statement (of ten; I can solve most problems if I invest the necessary effort) from the General Self-Efficacy Scale (GSE); 4-point scale: 1 (not at all true)– 4 (exactly true); (higher scores representing higher self-efficacy).	[31]
Perception of support^1^	Quality of support scale: One (of 3 validated subscales) from the COPE questionnaire (i.e Do you feel supported by your family; by health and social services): 4 of 15 questions; 4-point scale: 1 (never)– 4 (always); (higher scores representing higher perceived support), sums for each subscale	[32]
**Descriptive variables/ covariables**		
Demographics^1^	Age, gender, family caregiver’s relation to the patient	
Attachment security^1^	The Experiences in Close Relationships scale (ECR-M16); 16 items to measure attachment anxiety (fear of rejection and abandonment) and avoidance (discomfort with closeness and dependence on close others) in close relationships (including non-romantic partners); 7-point scale: 1 (lower attachment insecurity) - 7 (greater attachment insecurity)	[33]
Health-related quality of life^1^	The EuroQol-5D (EQ-5D), including five subscales: mobility, self-care, pain, usual activities, and psychological status; 3-point response scale: 1 (no problems) - 3 (severe problems). An index score was calculated for each respondent (–0.594 (worst possible health status)– 1.00 (best possible))	[34]
Patient symptom intensity^2^	The Edmonton Symptom Assessment System (ESAS)12 is a validated self-report tool. 9 common symptoms of advanced cancer (pain, tiredness, nausea, depression, anxiety, drowsiness, shortness of breath, appetite, well-being); 0–10 (higher scores representing worse symptom intensity). A total symptom distress score as a measure of overall symptom burden was calculated score (mean value of the nine symptoms).	[35]

^1^Reported by patients and caregivers

^2^Reported by patients.

### Statistical analysis

#### Similarity in dyad responses

For each of our five response variables, a Pearson correlation coefficient was used to assess covariation of answers within dyads, while group averages were compared with paired Student’s t-test. There were missing values, unequally distributed among the five response variables, mainly due to patients not completing questions (or being spared them by interviewer). This means that rather than 144 dyads, there were between 126 and 141 dyads in these analyses.

#### Dissimilarity within dyads

To highlight response variables for which the intra-dyad differences were large, a dataset was constructed based on the difference between the response given by the patient and that given by the family caregiver (patient minus family caregiver). Each dyad was then represented by one difference value per response variable; this was positive when the patient scored higher than the family caregiver and negative when family caregiver scored higher than the patient.

Each response variable was subjected to Generalised Linear Model (linear distribution, identity-link) with 13 independent variables (sociodemographic and clinical characteristics). We used a model selection approach in which the best model was chosen among all combinations of independent variables. The best model was selected using the Akaike information criterion (AIC), which considers both the explanatory power of the model and the number of independent variables. Even if our approach identifies the best model, there is often little support in claiming that all other top models are inferior. As our interest was in identifying the sociodemographic and clinical variables of relevance, we calculated how many models a variable was included in, then considering the top 200 models.

The variable”Relationship with patient” included two categories with too few respondents for meaningful analyses, “Sibling” (N = 3) and “Other relative/close friend” (N = 4), and those dyads were therefore excluded. Furthermore, these analyses can only be used for complete datasets, and thus dyads for which some of the sociodemographic and clinical data were missing were excluded meaning that less than 144 dyads were used (108 or 112 dyads per model). Most of the missing values stems from patients not completing questions (or being spared them by interviewer).

## Results

### Characteristics of the participants

The mean age (±SD) of the patients was 68.6 (±12.6) years and 54% were females. 97% of the patients had a cancer diagnosis (n = 139), 2% had COPD (n = 3) and 1% of the patients had ALS (n = 2). The mean age of the family caregivers was 62.3 (±13.0) years and 71% were spouses to the patient, 24% were children of the patient, others (5%) were siblings or a close friend. About half of the family caregivers (47%) were employed or self-employed, while 51% had disability pension/pension or were on sick leave (Table 2).

**Table 2:** Clinical and demographic characteristics of the dyads.

Characteristics	Patient (n = 144)	Family caregiver (n = 144)
Age, mean±SD	68.6±12.6	62.4±13.0
Female, n (%)	78 (54.2)	71 (50.0)
**Type of relation to the patient, n (%)**		
Husbound/wife	-------------	102 (70.8)
Child	-------------	35 (24.3)
Sibling	-------------	3 (2.1)
Other relative/close friend	-------------	4 (2.8)
**Living arrangements, n (%)**		
Married, n (%)	105 (72.9)	122 (84.7)
Children living in the same household	16 (11.6)	26 (19.8)
Living alone	39 (27.1)	17 (11.8)
Living in own accommodation	137 (95.4)	-------------
Living in caring home	5 (3.5)	-------------
**Education, n (%)**		
No formal education	8 (5.6)	6 (4.2)
Elementary school	38 (26.4)	29 (20.1)
Vocational education	34 (23.6)	31 (21.5)
High school	23 (16.0)	33 (22.9)
University	41 (28.4)	45 (31.3)
**Employment, n (%)**		
Employed or own company	-------------	67 (46.5)
Disability pension/ Pension	-------------	67 (46.5)
Sick leave	-------------	4 (2.8)
Unemployed	-------------	2 (1.4)
Parental leave	-------------	2 (1.4)
Other	-------------	2 (1.4)
**Medical diagnosis requires palliative care, n (%)**		
Cancer	139 (96.5)	-------------
COPD	3 (2.1)	-------------
ALS	2 (1.4)	-------------
Number of years having knowledge of the disease, mean±SD	4.1±3.8	

Patients perceived less stress than family caregivers, and higher perception of support, while the averages did not differ for the other three variables (Table 3).

**Table 3:** Patient and family caregiver scores on test variables including paired t-tests and correlations.

	Patient	Family caregiver	Paired t-test	Paired t-test	Correlation
	Mean (SD)	Mean (SD)	t-value	*p*-value	R (*p*-value)
Perceived stress (N = 127)	2.20 (0.91)	2.52 (1.01)	-2.86	0.0050**	0.148 (0.0980) NS
Self-efficacy (N = 126)	3.35 (0.636)	3.28 (0.689)	0.824	0.412	-0.077 (0.391) NS
Sense of security in care–Care interaction (N = 139)	5.17 (0.635)	5.17 (0.694)	-0.0250	0.980	0.243 (0.00396) **
Sense of security in care–Mastery (N = 141)	4.26 (1.105)	4.32 (0.969)	-0.537	0.592	0.226 (0.00702) **
Perception of support (N = 126)	13.76 (2.14)	12.63 (2.80)	4.58	0.000011***	0.390 (0.000006) ***

* p< 0.05

** p< 0.01

*** p< 0.001.

### Similarity pattern of perceptions within dyads

There were significant positive correlations between the responses given by the patient and those given by the family caregiver for three of the five variables (“Perception of support”, “Sense of security in care–Mastery” and “Self-efficacy”) (Table 3).

### Dissimilarity patterns of perceptions within dyads

When searching for dissimilarity patterns using model selection, two cautionary principles should be applied. First, any model with weak support–i.e. large *p*-value; partial regression coefficients being NS; explanatory variables inconsistently included in the top 200 models–should not be used to draw in-depth conclusions. There is simply a high risk that alternative top models might include other explanatory variables. Second, explanatory variables not consistently included in the top 200 models, are also subject to the same risk that alternative top models might exclude it. On the other hand, models with strong support–i.e. small *p*-value; with significant partial regression coefficients; and explanatory variables consistently included in the top 200 models–are much better suited for detailed study.

For the above reasons, we focus mainly on two models with very strong support, i.e. differences within the dyads could be explained by one or more sociodemographic and clinical characteristics: “Perceived stress” and “Perception of support”. In contrast, “Sense of security in care–Care interaction” resulted in a barely significant model and its two partial regression coefficients were NS (Table 4). The model selected for “Self-efficacy” was clearly significant but only two of its four partial regression coefficients were significant and only one–patient symptom severity–was consistently selected among the top 200 models (Table 4). Inspecting the data, this was due to patient “Self-efficacy” going down with symptom severity while that of family caregivers being unaffected (S1 File). The selected model for “Sense of security with care–Mastery” was also clearly significant, but resulted in a model that was rather complex, with five explanatory variables that were selected with relatively low support (most occurring in less than 90% of the top models, and partial regression coefficients being non-significant or weakly significant). However, family caregiver genus had relatively strong support: male family caregivers scored higher than female while patients scored lower when caregiver was male (S2 File).

**Table 4:** The best selected model for each the five response variables under study.

	Perceived stress (N = 112)	Self-efficacy (N = 112)	Sense of security in care–Care interaction (N = 108)	Sense of security in care–Mastery (N = 112)	Perception of support (N = 112)
P of selected model	0.000000	0.008172	0.024113	0.003103	0.000066
Patient Gender: female^1^		-1.45 NS (47%)	+1.93 NS (68%)		
Family caregiver Gender: female^1^	-4.16 *** (100%)			+2.35 *(94%)	
Family caregiver Relation: Child^2^					+4.39 *** (97%)
Patient Age				-2.19 * (77%)	-3.31 *** (99%)
Family caregiver Age		-2.06 * (70%)			
Patient Attachment Anxiety	+1.56 NS (47%)				
Family caregiver Attachment Anxiety	-2.26 * (88%)		+1.98 * (92%)	+2.03 * (86%)	
Patient Attachment Avoidance					
Family caregiver Attachment Avoidance					+2.14 *(78%)
Patient HRQoL^3^	-2.15 * (69%)				-2.43 * (90%)
Family caregiver HRQoL^3^	+3.29 *** (100%)	-1.52 NS (59%)		-1.65 NS (65%)	-1.60 NS (56%)
Patient symptom severity		-3.06 ** (100%)		-2.21 * (74%)	
Excluded variable with the highest occurrence among top 200 models	56%	33%	50%	29%	36%

^1^Compared with male

^2^Compared with Husband/Wife

^3^HRQoL = Health-related Quality of Life.

* p< 0.05

** p< 0.01

*** p< 0.001; NS non-significant.

Response variables were created as the difference between patient and family caregiver responses. Values are Z-values, significance, and the percentage of the top 200 models that included the variable (within parentheses).

It is important to keep in mind that the response variables are based on differences, and that they represent gradients from patients perceiving lower degrees than the family caregiver (negative values), to patients perceiving higher degrees than the family caregiver (positive values). To stress this complex feature of the response variables, we call them “within-dyad gradient in perceived stress”, etc, in the results section below.

*Within-dyad gradient in perceived stress*. The model selected included five independent variables, three of these were frequently (>88%) included in the top 200 models. The gradient of perceived stress (i.e. the difference going from family caregiver perceiving higher stress than patients, to patients perceiving higher stress) was positively related to increasing levels of “Family caregiver HRQoL” (Table 4). In contrast, there was a negative relationship between the within-dyad gradient in perceived stress and higher levels of “Family Caregiver Attachment Anxiety” (Table 4). Furthermore, the response variable was lower when the family caregiver was female compared with when the caregiver was male (Table 4); patients perceived less stress than the family caregiver when the latter was female (mean difference in perceived stress -0.71; CI_95%_ -1.034, -0.385; N = 62) while there was no difference in perceived stress when the family caregiver was male (0.095, CI_95%_ -0.184, 0.375, N = 63).

*Within-dyad gradient in perception of support*. The model selected included five independent variables, three of these were frequently (>90%) included in the top 200 models. This response variable was higher when the family caregiver was a child of the patient (Table 4; mean difference 2.33, CI_95%_ 1.24, 3.42, N = 30) compared to when the family caregiver was a husband or wife (0.83, CI_95%_ 0.27, 1.39, N = 90). In contrast, this response variable decreased with “Patient Age” (Table 4); younger patients perceived higher degrees of support than family caregivers while they reported similar perception when patients were old (S1 Data). A negative relationship was also seen between this response variable and “Patient HRQoL” (Table 4); patients with low quality of life perceived higher degrees of support than the family caregiver while their perception was similar at high quality of life (S1 Data).

## Discussion

This study provides new insights about the dyads of patients in palliative care and their closest relative. It showed how they, within the dyads, experience similar and dissimilar pattern of stress, self-efficacy, dimensions of sense of security and perceived support and in what way these patterns are associated with aspects such as family caregiver gender, relationship, patient age, attachment styles and health-related quality of life.

Within the dyads, the patient and the family caregiver scores showed a similar pattern in three of the five variables assumed to be important for care, namely their “Sense of security in care–Care interaction”, “Sense of security in care–Mastery”, and “Perception of support”. In contrast, their scores for “Self-efficacy” were not correlated and “Perception of stress” displayed a weak but non-significant correlation. Previous research supports these findings and has shown that patient-family caregiver dyads often agree well in their ratings of the perceived reality of quality of care [36,37] and stress [38]. Our findings regarding self-efficacy need further study.

Further, within-dyad dissimilarities that could be attributed to sociodemographic or clinical characteristics were found in the responses regarding “Perceived stress” and “Perception of support”. One of the main findings was that when the patient scored a higher level of stress than the family caregiver, the family caregiver (in the dyad) was more likely to be male. In addition, male gender of the family caregiver was associated with patients scoring lower levels than the family caregiver in “Sense of security in care–Mastery”. There are several possible explanations of the results. As the patients’ cancer progresses and they become more ill, they require more care and support from the family caregiver [39,40]. The mean age of the caregivers was 63 years, and thus they may have been subject to traditional generational gender differences in which caregiving for children and relatives is mainly performed by females. Also, it is less likely that older men have had caring occupational roles in their working life. However, when inspecting the data in more detail, gender affected the difference for “Perceived stress” mainly by female family caregivers scoring much higher than male ones (S3 File).

The finding that patients with female family caregivers experienced less stress than those with male family caregivers agrees with the findings of two previous studies [37,38], although a third study failed to detect such a difference [36]. It is possible that female gender of the family caregiver decreases the risk *per se* of perceiving higher stress and lower mastery in the caregiving situation. However, some previous research found no such gender difference regarding stress [36], while others found that females reported more stress than men, regardless of whether they were patients or family caregivers [37,38]. Since high levels of stress are related to poor outcomes, it is important to intervene to decrease the levels of distress. A recent review examined how group therapy, including Supportive‐Expressive Group Therapy and Cognitive‐Existential Group Therapy effects the levels of stress, anxiety and depression [41]. The authors found no support for these interventions, but there were some methodological issues in a few studies. When these studies were excluded, there was support for group therapy interventions can lower the experience of stress, anxiety and the occurrence of depression. The authors concludes that the link between existential stressors, adjustment processes, and existential distress patterns needs to be further studied [41]. According to our results, we suggest to also consider gender as an important aspect in further research examining the effect of different forms of group therapy.

The family caregiver was more likely to be a child of the patient (than a spouse) in dyads in which the patient scored a higher level of support than the family caregiver. Several training modules designed to improve the caring skills of healthcare professionals for patients and family caregivers have been tested [42,43]. These do not, however, provide direction in how to support children or relatives of the patient who are not living with the patient (which is the case with most children in our study). It is probable that the caring needs differ between family caregivers living together and those not living together with the patient, since persons who do not live in the same household meet the healthcare professionals less often and, in this way, have less opportunity to receive guidance and support. We suggest that individualised and more targeted interventions that address both practical and mental components will improve outcomes. Such interventions should consider gender aspects and identify the care needs of patients’ children when they take on a caregiving role.

Another variable that also had high explanatory power regarding dissimilarities within patient-family caregiver dyads was attachment anxiety, and especially that of the family caregiver. The psychological stress of cancer patients does not differ from stress perceived by their family caregivers [5], and the attachment systems of patients and family caregivers are activated as a response to stress. Insecure attachment styles may interfere with the way that individuals cope with a challenging situation, such as being in the cancer context. For example, demoralisation in patients with cancer is associated with lower attachment security (with a stronger effect for attachment anxiety) [44], and both patients with cancer and family caregivers who score high levels of attachment anxiety are at the risk of experiencing higher levels of depressive symptoms and anxiety, and of perceiving poor social support [45]. Overall, these findings suggest that attachment styles are relevant to the psychological adjustment of long‐term cancer survivors, which includes their experience of negative cancer‐specific outcomes [46]. Our results also support previous findings that the attachment style of a patient or family caregiver may be related not only to the way in which the individual copes with the situation, but also to the way in which the other part of the dyad copes. For example, the attachment style of a caregiver can influence how they respond to a patient’s needs, and attachment anxiety or avoidance is more likely to interfere with effective and sensitive caregiving than secure attachment [6]. Our results suggest that dissimilarities within the way in which the parties of a dyad score “Perceived stress” are linked to insecurity in attachment style. We conclude, therefore, that healthcare staff must consider the attachment patterns of both the patient and the family caregiver. The attachment style of the family caregiver may become a stressor for the patient, and *vice versa*, which affects their adaptation to changes brought on by cancer or other severe diseases.

Moreover, we found that younger patients more often scored higher levels than the family caregiver for “Perception of support”. There is a complex pattern related to patient age, with younger patients being at a higher risk for patient anxiety, while caregiver anxiety is not related to age [47]. In addition, the risk of severe grief and depression after the patient’s death is higher when caring for younger or middle-aged cancer patients [48]. In our data however, “Perception of support” among patients decreased with age, while the perception among family caregivers was unaffected by patient age (S4 File).

Finally, “Perception of support” is a significant contributor to patient’s HRQoL. Patients who less frequently sense family support experience stress more often and perceive general well-being less often than those with a strong sense of family support [49]. Lack of support from family and friends is a risk factor for increased symptom severity, while impaired physical function is associated with increased stress and a poor QoL [50]. Our data showed a slight decrease of “Perception of support” with increasing quality of life among patients (S5 File), possibly reflecting a lower supportive need when HRQoL is higher. In contrast, “Perception of support” of family caregiver increased with patients’ quality of life (S5 File).

### Limitations

Our study suffers from some limitations. First, the design was cross-sectional, and therefore the associations that we have observed may not be causal. Second, multiple testing may contribute to type I error, i.e. the rejection of a true null hypothesis (a “false positive” finding). However, our approach was mainly exploratory, using model selection techniques where P-values can help to support interpretation rather than strictly testing null hypotheses. Thirdly, although 65% of the eligible patients were interviewed (174/267), which we consider relatively high for a study of this type, in which patients with disease in a palliative stage are interviewed, those who chose to participate differed from those who declined (with the participants being younger than the decliners). Of the 174 patients interviewed, 144 (83%) had a family caregiver who also participated in the study. This may have consequences for whether conclusions drawn from the results can be generally applied. There are, of course, other potential confounding factors, like the duration since diagnosis (in our case up to 8 years) or health status of the caregiver (average age 62 years, and about half being employed, both suggesting an acceptable caregiver health status in many dyads). Data was collected in 2009–10. Since that time period, there has not been any large changes regarding the organisation of the palliative care. Possibly it has expanded to patients with non-malignant diseases, and the length of stay in hospital has become even shorter since the data were collected. This makes it more important to recognise patients’ and family members’ needs in the palliative homecare context.

## Conclusions and clinical implications

To attain the goals of palliative care, health professionals are encouraged to offer both the patient and the family caregiver individualised practical and emotional support. The within dyadic interaction must be considered when providing such support. Recent research has shown some promising results that an increase in dyadic coping may reduce stress in patients with advanced cancer and their family caregiver caregivers in specialised palliative care [51].

Our study has shown that the patient and the family caregiver within a dyad often have similar pattern of perceptions (in three of the five variables). It is, however, important that the healthcare staff identify when the perceptions within the dyad differ regarding “Perceived stress” and “Perception of support”. This is necessary if healthcare staff are to recognise the unique needs of patients and family caregivers in different situations, provide adequate support, and facilitate dyadic coping.

We have also presented new insights into within-dyad associations: gender, age and attachment style affect such associations in responses regarding differences in “Perceived stress” and “Perception of support”. In addition, family caregivers who are children of the patient are at risk of perceiving less support than the patient, which may be important for the healthcare staff to recognise.

## Supporting information

S1 Data (XLSX)

S1 File Self-efficacy and patient symptom severity among patients and family caregivers. (TIF)

S2 File Sense of security with care–Mastery and family caregiver genus. (TIF)

S3 File Perceived stress and family caregiver genus. (TIF)

S4 File Perception of support and patient age. (TIF)

S5 File Perception of support and patients HRQoL. (TIF)
