# Supplementary figures and images for: Dying within dyads: Stress, sense of security and support during palliative home care
Source: PLoS One. 2021 Sep 14;16(9):e0257274. doi: 10.1371/journal.pone.0257274 (PMC8439476; doi:10.1371/journal.pone.0257274)

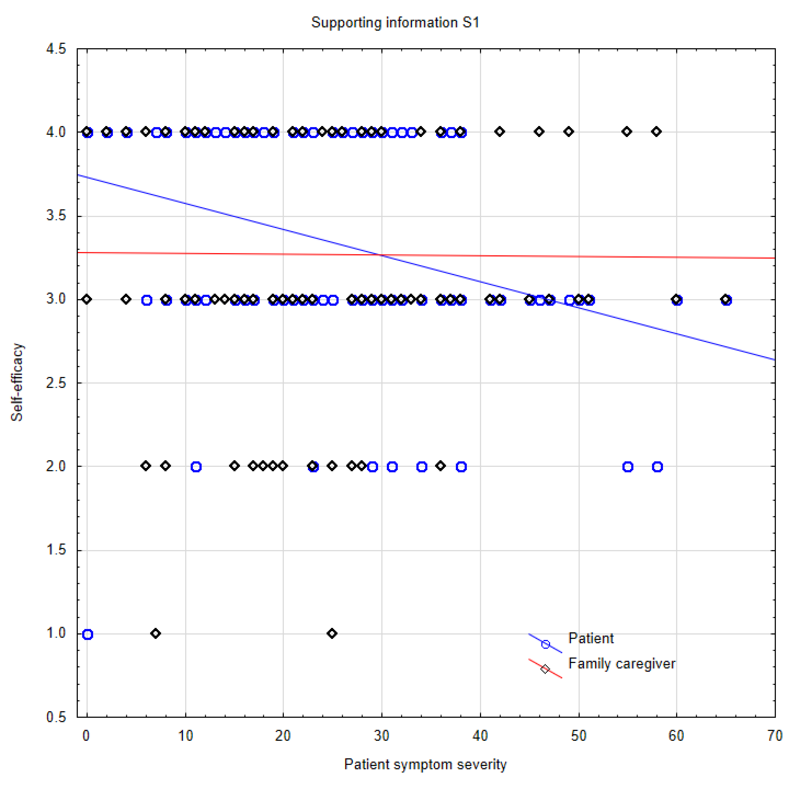

Supplement: S1 File — (TIF) [file pone.0257274.s002.tif]

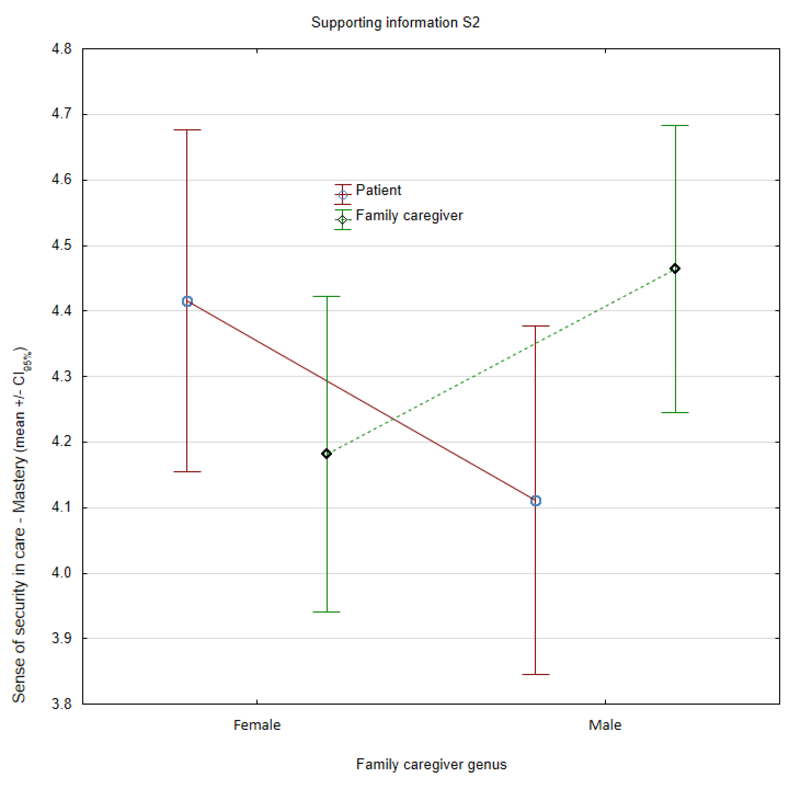

Supplement: S2 File — (TIF) [file pone.0257274.s003.tif]

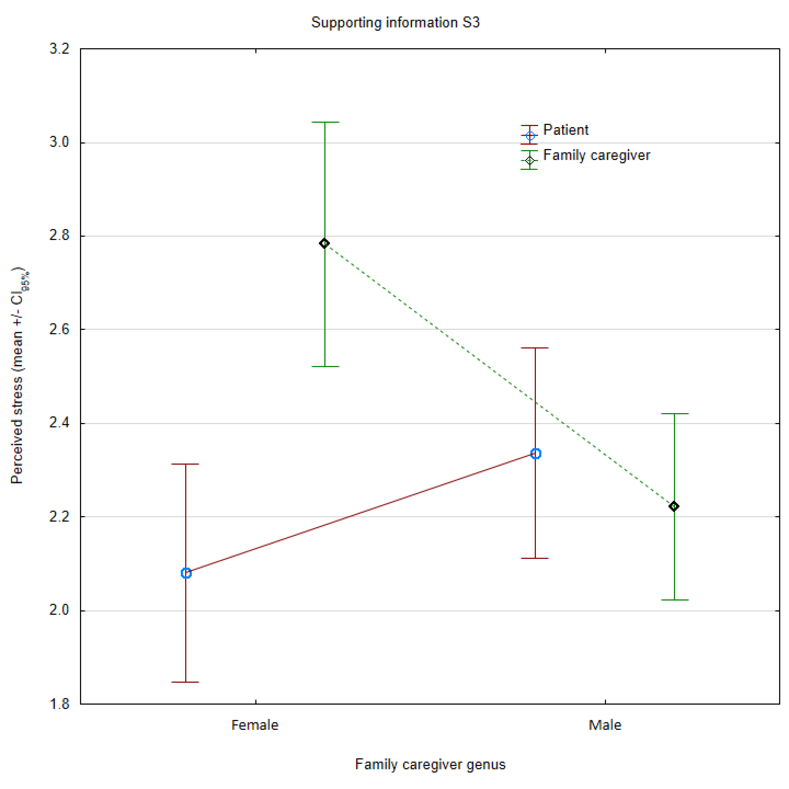

Supplement: S3 File — (TIF) [file pone.0257274.s004.tif]

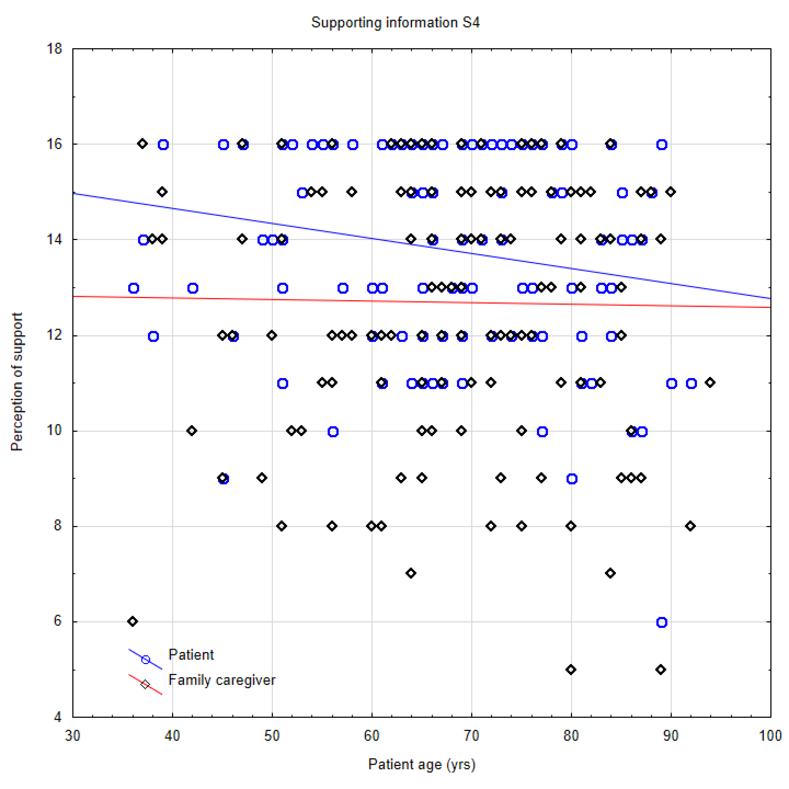

Supplement: S4 File — (TIF) [file pone.0257274.s005.tif]

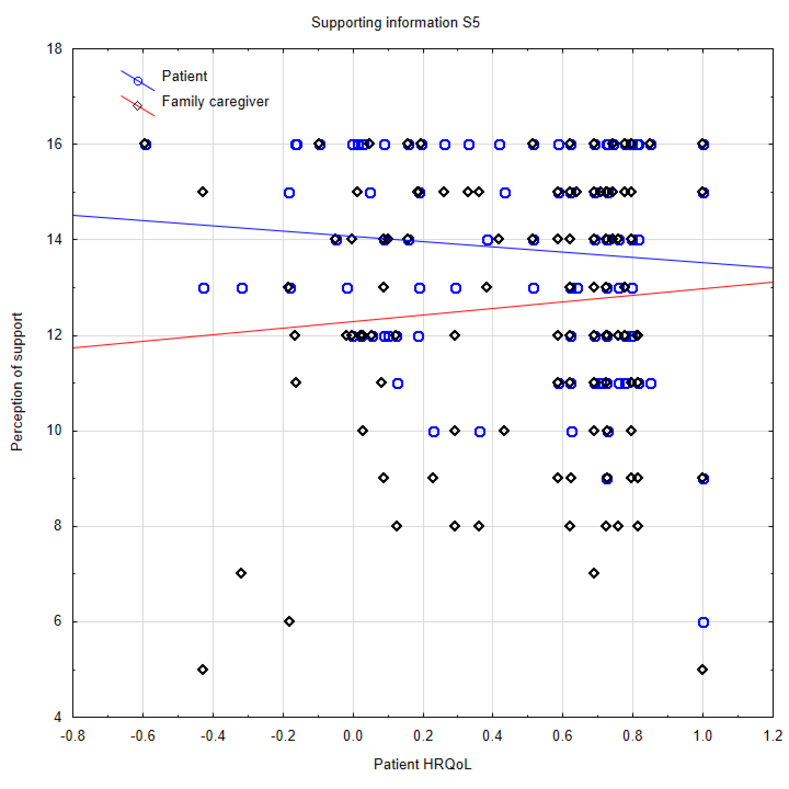

Supplement: S5 File — (TIF) [file pone.0257274.s006.tif]
